# Effect of Chronic Social Defeat Stress on the Small-Intestinal Environment, Including the Gut Flora, Immune System, and Mucosal Barrier Integrity

**DOI:** 10.3390/ijms26199359

**Published:** 2025-09-25

**Authors:** Soichi Yagi, Hirokazu Fukui, Tetsuya Shiraishi, Koji Kaku, Midori Wakita, Yasuhiro Takagi, Maiko Ikenouchi, Toshiyuki Sato, Mikio Kawai, Yoko Yokoyama, Tetsuya Takagawa, Toshihiko Tomita, Shiho Kitaoka, Shinichiro Shinzaki

**Affiliations:** 1 Department of Gastroenterology, Hyogo Medical University School of Medicine, 1-1 Mukogawa, Nishinomiya 663-8501, Japan; so-yagi@hyo-med.ac.jp (S.Y.); te-shiraishi@hyo-med.ac.jp (T.S.); ds23003@hyo-med.ac.jp (K.K.); mi-wakita@hyo-med.ac.jp (M.W.); ya-takagi@hyo-med.ac.jp (Y.T.); ma-ikenouchi@hyo-med.ac.jp (M.I.); tshnngn@hyo-med.ac.jp (T.S.); mkawai@hyo-med.ac.jp (M.K.); yoko0502@hyo-med.ac.jp (Y.Y.); tomita@hyo-med.ac.jp (T.T.); sh-shinzaki@hyo-med.ac.jp (S.S.); 2 Center for Clinical Research and Education, Hyogo Medical University, Nishinomiya 663-8501, Japan; takagawa@hyo-med.ac.jp; 3 Department of Pharmacology, Hyogo Medical University School of Medicine, Nishinomiya 663-8501, Japan; sh-kitaoka@hyo-med.ac.jp

**Keywords:** psychological stress, small intestine, gut microbiota, mucosal barrier, microinflammation

## Abstract

Psychological stress is deeply involved in the pathophysiology of gastrointestinal diseases. We investigated the effect of psychological stress on the small-intestinal environment, including gut flora, immune system, and mucosal integrity in mice subjected to chronic social defeat stress (CSDS). CSDS mice were established by exposing a C57BL/6N mouse to an ICR aggressor mouse. Stool samples were obtained to investigate its properties and the gut microbiome profile. Using small-intestinal tissues, the expression of cytokines, antimicrobial peptides, and tight junction proteins (TJPs) were examined by real-time RT-PCR and immunohistochemistry. Small-intestinal permeability was evaluated by transepithelial electrical resistance assay. For stool properties, mean Bristol scale score and fecal water content were significantly lower in the CSDS group. *Pseudomonadota* and *Patescibacteria* were significantly more abundant in the stools from CSDS mice. Among TJPs and antimicrobial peptides, the expression of Occludin, Claudin-4, and Regenerating gene IIIγ was significantly decreased in the small intestine epithelium of CSDS mice. The small-intestinal permeability was significantly increased in CSDS mice. Lipopolysaccharide immunoreactivity, the number of macrophages, and proinflammatory *IL-1β* expression were significantly increased in the small intestine of CSDS mice. These findings suggest that psychological stress is associated with mucosal barrier dysfunction and microinflammation in small-intestinal tissues.

## 1. Introduction

There is much evidence to suggest that psychological stress greatly affects not only gastrointestinal physiology but also the immune system [1,2,3]. Studies using several animal models have shown that psychological stress causes visceral hypersensitivity and/or dysmotility [4,5], and similarly, visceral hypersensitivity and dysmotility are considered to be central to functional gastrointestinal disorders in human patients with frequent mental disturbance [6,7]. Furthermore, it is known that patients with depression frequently suffer from constipation [8,9]. In patients with depression, recent studies have shown that the intestinal environment, including gut flora, immune system, and mucosal barrier function, is disrupted, affecting gastrointestinal physiology [10,11]. However, much remains unclear about the role of psychological stress in the pathophysiology of the gastrointestinal tract.

It is well known that the gut and brain show close physiological interactions, and that numerous molecules, including gut hormones, short-chain fatty acids, bile acids, antimicrobial peptides, and cytokines, play pivotal roles as mediators in the so-called brain–gut axis [12,13]. The profiles of these mediators are orchestrated by gut flora and the small-intestinal immune system, maintaining intestinal mucosal integrity [14] and protecting the host from pathogens and harmful antigens [15]. However, little is known about the effect of psychological stress on the small-intestinal immune system and its associated pathophysiology in terms of defecation.

Chronic psychological stress plays a pivotal role in the development of depression [16,17]. Since chronic social defeat stress (CSDS) effectively accelerates the development of depression [18,19], a mouse model exposed to CSDS is widely used to investigate the effect of psychological stress on not only the brain but also the gastrointestinal tract [20,21]. Interestingly, a few studies have shown that the alterations of gut flora and fecal metabolites in CSDS mice are consistent with those in patients with depression [22,23]. Therefore, in the present study, we comprehensively examined the effect of psychological stress on the defecation-associated intestinal environments, including the gut microbiome and intestinal immune system in a depression model using mice subjected to chronic social defeat stress (CSDS).

## 2. Results

### 2.1. Social Interaction Test in CSDS Mice

**Figure 1:**
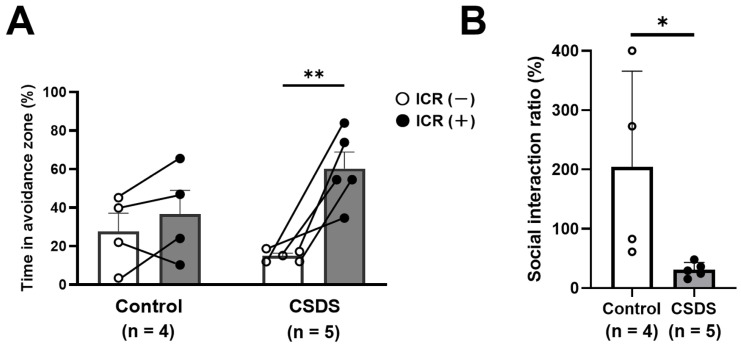
(**A**) Time spent in the avoidance zone in the absence or presence of the Institute of Cancer Research (ICR) aggressor mouse. (**B**) The social interaction ratio was calculated by dividing the time spent in the social interaction zone in the presence of the ICR aggressor mouse by the time spent in its absence. Significantly different between the two situations with and without the ICR aggressor mouse: * *p* < 0.05; ** *p* < 0.01 vs. control group. CSDS, chronic social defeat stress.

The social interaction test was performed to evaluate the effect of CSDS on the development of social avoidance. The time spent in the avoidance zone did not differ between the situations with and without the Institute of Cancer Research (ICR) aggressor mouse in the control group (Figure 1A). On the other hand, it was significantly longer in the CSDS group when the ICR mouse was present (60.3 ± 8.6%) than when it was absent (15.0 ± 1.4%). Furthermore, the social interaction ratio was significantly lower in the CSDS group (Figure 1B).

### 2.2. Stool Properties in Mice Subjected to CSDS

**Figure 2:**
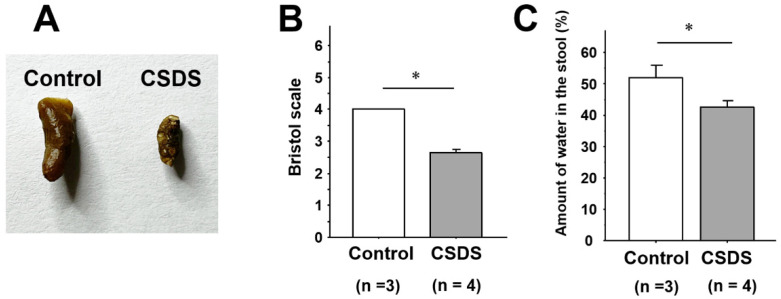
Stool properties of mice with chronic social defeat stress. (**A**) Appearance of stools. (**B**) Stool form according to the Bristol scale. (**C**) Stool water content. * *p* < 0.05 vs. control group. CSDS, chronic social defeat stress.

The appearance of the stools was scored according to the Bristol scale [24]. The feces of the control mice were all normal in shape and moisture content (Bristol scale score 4.0 ± 0.0) (Figure 2A,B). In the CSDS group, however, the Bristol scale score was significantly lower (2.6 ± 0.1), suggesting a reduction of stool moisture (Figure 2A,B). Indeed, stool water content in the stool was significantly lower in the CSDS group (42.6 ± 3.5%) than in the controls (56.9 ± 3.4%) (Figure 2C).

### 2.3. Effect of CSDS on the Small-Intestinal Gut Flora

**Figure 3:**
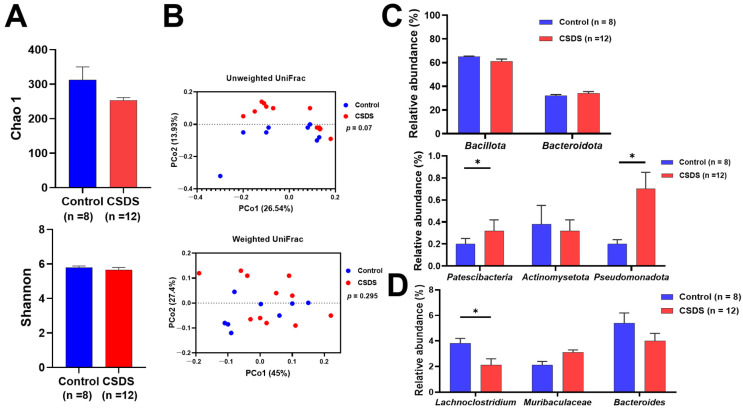
Effect of chronic social defeat stress on fecal microbiota. (**A**) Chao-1 and Shannon diversity indices indicate α-diversity of the gut microbiota. (**B**) The β-diversity of the gut microbiota. The relative abundance of small-intestinal bacteria at (**C**) the phylum and (**D**) the genus levels. * *p* < 0.05 vs. control group. CSDS, chronic social defeat stress.

We next investigated alterations of the gut flora by examining the stools of the experimental mice. The Chao-1 and Shannon diversity indices for α-diversity assessment did not differ between the control and the CSDS mice (Figure 3A). The assessment for β-diversity showed no significant difference between the control and the CSDS mice (Figure 3B). Furthermore, we investigated the phylum and genus profiles of the gut microbiome in these mice. At the phylum level, *Patescibacteria* and *Pseudomonadota* were significantly increased in the CSDS mice (Figure 3C). On the other hand, at the genus level, *Lachnoclostridium* was significantly decreased in the CSDS mice (Figure 3D).

### 2.4. Effect of CSDS on Tight Junction Proteins Expression, Morphology and Permeability in the Small-Intestinal Mucosa

**Figure 4:**
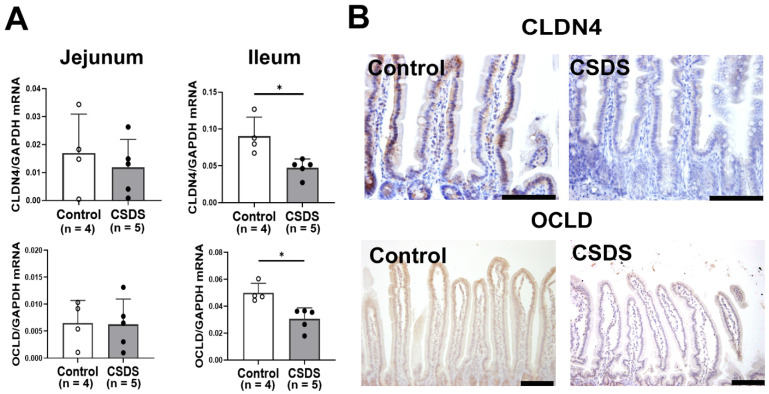
(**A**) Effect of chronic social defeat stress on mRNA expression of tight junction molecules in the small intestine. (**B**) Representative images of immunostaining for CLDN4 and OCLD in the small-intestinal mucosa. * *p* < 0.05 vs. control group. CLDN4, Claudin-4; OCLD, Occludin. CSDS, chronic social defeat stress. Bar = 100 μm.

Furthermore, the expression of tight junction proteins (TJPs) in the small-intestinal tissues was examined in the experimental mice. The expression of *Claudin-4* and *Occludin* mRNAs was significantly decreased in the ileum of the CSDS mice (Figure 4A). Consistent with these findings, immunohistochemistry showed that both Claudin-4 and Occludin expressions were also decreased in the small-intestinal epithelium of the CSDS mice compared with the controls (Figure 4B).

**Figure 5:**
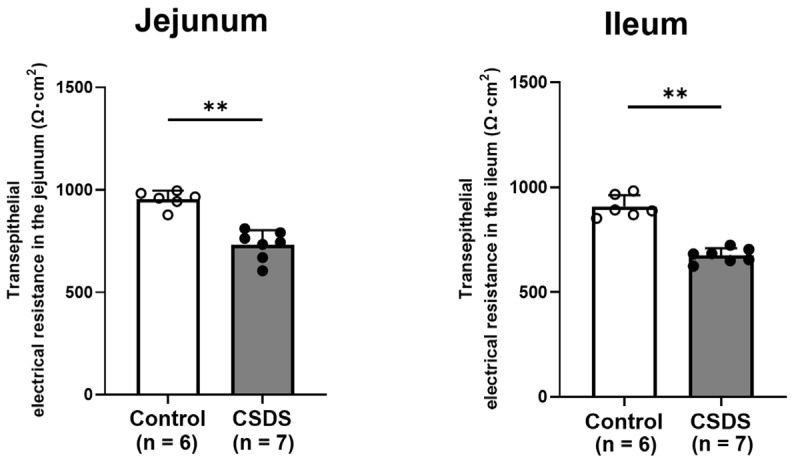
Effect of chronic social defeat stress on permeability of the small-intestinal mucosa. ** *p* < 0.01 vs. control group. CSDS, chronic social defeat stress.

The transepithelial resistance values in the small-intestinal tissues were significantly lower in CSDS mice than in the controls (Figure 5). The height of villi was significantly lower in CSDS than in control mice, although the depth of crypt did not differ between the two groups statistically. However, the ratio of villi to crypt was subsequently smaller in CSDS than in control mice (Supplementary Figure S1).

### 2.5. Expression of Antimicrobial Peptides, Cytokines, LPS, and CD80 in the Small Intestine of CSDS Mice

**Figure 6:**
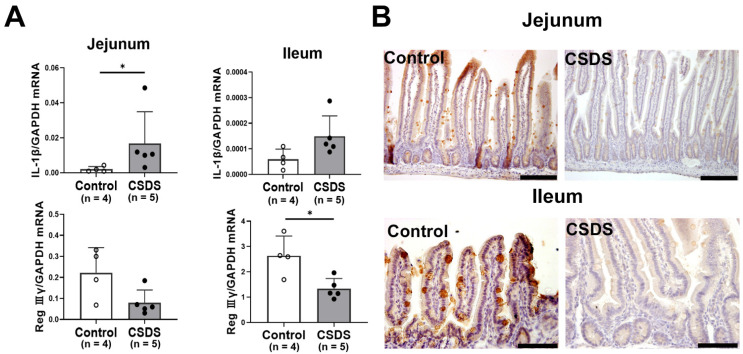
(**A**) Effect of chronic social defeat stress on mRNA expression of cytokines and antimicrobial peptide Reg Ⅲγ in mouse small-intestinal tissues. (**B**) Immunostaining for Reg Ⅲγ in the small-intestinal mucosa. * *p* < 0.05 vs. control group. CSDS, chronic social defeat stress. Bar = 100 μm.

We further investigated the expression of antimicrobial peptides and cytokines in the small intestine of the experimental mice. The mRNA expression of *Interleukin-1*β (*IL-1*β) was significantly increased in the jejunum and tended to be higher in the ileum of the CSDS mice (Figure 6A). Among anti-microbial peptides, the mRNA expression of *Reg IIIγ* was significantly decreased in CSDS mice relative to the controls in the jejunum and ileum (Figure 6A). Indeed, Reg Ⅲγ was apparently decreased in the CSDS mice. (Figure 6B). 

**Figure 7:**
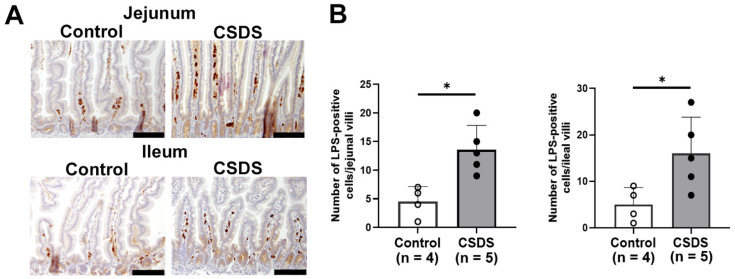
Effect of social defeat stress on LPS immunoreactivity in the small-intestinal tissue. (**A**) Immunostaining for LPS in the small-intestinal mucosa. (**B**) Graphs showing the number of LPS-positive cells in the small-intestinal mucosa. * *p* < 0.05 vs. control group. CSDS, chronic social defeat stress. Bar = 100 μm.

To examine changes in harmful antigens in the small-intestinal mucosa, we examined the immunoreactivity of LPS using immunohistochemistry. In both the jejunum and ileum, strong LPS immunoreactivity was evident in the lamina propria of the small intestine in CSDS mice relative to the controls (Figure 7A). The number of LPS-positive cells was also significantly increased in the jejunum and ileum of CSDS mice relative to the controls (Figure 7B).

**Figure 8:**
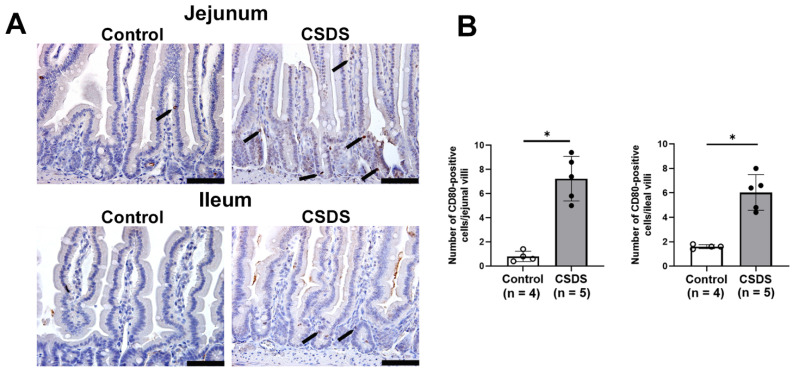
Effect of social defeat stress on CD80 immunoreactivity in the small-intestinal tissue. (**A**) Immunostaining for CD80 in the small-intestinal mucosa. (**B**) Graphs showing the number of CD80-positive cells in the small-intestinal mucosa. * *p* < 0.05 vs. control group. CSDS, chronic social defeat stress. Arrows indicate CD80-positive cells. Bar = 100 μm.

Moreover, we found that CD80-positive cells were increased in the lamina propria of the small intestine in CSDS mice relative to the controls (Figure 8A). As shown in Figure 8B, the number of CD80-positive cells was significantly increased in the jejunum and ileum of CSDS mice relative to the controls (Figure 8), suggesting that immune cells such as macrophages were increased in the latter.

## 3. Discussion

Accumulating evidence suggests that psychological stress alters the intestinal environment [25,26], although the underlying pathogenesis remains unclear. Multiple depression models (chronic unpredictable mild stress, chronic restraint stress, CSDS or learned helplessness, etc.) have been used to investigate the pathophysiology in patients with major depressive disorder [27]. However, major depressive disorder is considered to be a heterogeneous disease [28], and, therefore, it seems very difficult to establish a perfect animal model for major depressive disorder. In the present study, although we selected a CSDS model, this model cannot avoid physical stress, and its behavior phenotypes are various according to the differences in protocol [29,30]. Therefore, in order to assess the effect of psychological stress on the intestinal environment, we first isolated the only mice susceptible to CSDS using a social interaction test and used them for the subsequent experiment. Subsequently, we found a significant reduction of the Bristol scale for fecal humidity in CSDS mice and indeed confirmed a significant decrease of the stool water content in those mice. These findings are compatible with clinical evidence that patients with depression frequently suffer from constipation [8,31], suggesting that CSDS mice are a useful model for investigating the pathophysiology of depression-associated constipation. 

The luminal environment of the intestinal tract plays a key role in the interaction between the gut and the brain [32]. There is now considerable evidence to suggest that an imbalance of the gut microbiome profile (dysbiosis) plays an important role in not only inflammatory diseases but also functional disorders of the gastrointestinal tract [33,34]. Moreover, dysbiosis has been associated with psychological stress [35,36], and in the present study, we were able to show that *Patescibacteria* and *Pseudomonadota* were significantly increased in the CSDS mice. Although the role of *Patescibacteria* in human diseases is not fully understood, it represents a diverse group of bacteria accounting for a significant proportion of microbial dark matter [37] and is frequently detected in various environments, including groundwater, freshwater, and the human oral cavity [38,39,40]. Interestingly, *Pseudomonadota* is also reported to be a predominant phylum in the oral cavity [41] and associated with gut dysbiosis and chronic intestinal inflammation [42,43]. It was noteworthy that the expression of proinflammatory IL-1β was consistently enhanced in the small-intestinal tissues of CSDS mice. It has often been debated whether dysbiosis is a pathophysiological cause or a consequence of specific diseases. Although various studies have reported dysbiosis in patients with constipation and/or depression, the candidate microbiota isolated varied considerably [44,45,46]. For instance, even in a similar CSDS model established by our group [47], the gut flora data obtained were quite different from those in the present study, suggesting that subtle differences in experimental conditions are likely to alter the gut flora. In this context, we propose that the dysbiosis observed in our CSDS model is a consequence rather than a cause.

The mucosal barrier of the intestine is crucial for protecting the host from invasion by harmful antigens or pathogens [48,49]. It has recently been highlighted that disruption of mucosal barrier function is an important first step in alteration of innate immunity, triggering microinflammation of the gastrointestinal mucosa that may be linked to altered pathophysiology in other organs [50,51]. Indeed, in the present study, we found that expression of tight junction proteins in the small intestine and mucosal permeability were enhanced in CSDS mice. These findings suggest that CSDS mice are likely to have disturbed small-intestinal mucosal barrier function. Furthermore, we found that antimicrobial Reg IIIγ was down-regulated in the small-intestinal mucosa, suggesting that host defense against pathogens may be inhibited in CSDS mice. Together, these changes in the environment of the small intestine might make it easier for harmful antigens/pathogens to invade. Compatible with these findings, the expression of LPS immunoreactivity was augmented in the small-intestinal mucosa, and the number of macrophages was also increased in the CSDS mice. Overall, psychological stress may disturb intestinal mucosal barrier function by inhibiting the expression of tight junction proteins and antimicrobial peptides, resulting in activation of proinflammatory immune cells against harmful antigens such as LPS.

Many physiological functions, including appetite, gastrointestinal motility, emotion, visceral sensation, and gut hormone secretion, are strictly controlled via the gut–brain axis. Regarding the link between gut environmental factors and the central nervous system, recent studies have suggested that gut microbiome-related metabolites (short-chain fatty acids, bile acids, vitamins, gut hormones, etc.) mediate gut to brain communication [45,52]. On the other hand, it remains unclear how psychological stress disturbs intestinal mucosal barrier function. In this context, Schneider et al. have recently proposed a mechanism whereby psychological stress induces inflammatory enteric glia, and that enteric neuron immaturity can lead to delayed intestinal transit time and monocyte-mediated inflammation in the gastrointestinal tract [53]. Although their stress model differs from ours, it is interesting that similar features, such as microinflammation and constipation, were observed in both. Since we did not examine the behavior of enteric neurons, we are yet unable to consider the extent to which the enteric nervous system (ENS) played a role in intestinal motility and inflammation in CSDS mice. However, in terms of increased intestinal mucosa permeability, it may be interesting to investigate how psychological stress affects the expression of tight junction proteins and antimicrobial peptides in the intestinal epithelial cells via ENS alteration.

In summary, we have shown that CSDS alters the flora and stool properties of mice. Furthermore, mice subjected to CSDS showed increased expression of tight junction proteins (Claudin-4 and Occludin) and microbial peptide Reg IIIγ in the small intestine and increased permeability of the intestinal mucosa. In addition, these mice showed an increase in the macrophage population and higher expression of proinflammatory IL-1β in the small-intestinal mucosa, in association with augmented LPS immunoreactivity. Although the underlying links between these changes remain unclear, we speculate that psychological stress led to mucosal barrier dysfunction, subsequently promoting the invasion of harmful antigens such as LPS, and resulting in microinflammation of the intestinal mucosa. As the ENS certainly plays a pivotal role in brain–gut communication [53], future studies will need to clarify the mechanism by which psychological stress acts on the ENS to disturb mucosal barrier function and intestinal motility.

## 4. Materials and Methods

### 4.1. Animal Model of Chronic Social Defeat Stress

C57BL/6N mice (male; 9 weeks old) and ICR mice (male; 9 weeks old) were purchased from Japan SLC. The mice were kept under specific pathogen-free conditions in a controlled clean room (temperature 23 ± 1 °C; humidity, 50 ± 5%) with a 12 h light/12 h dark regime and allowed free access to laboratory food (Oriental Yeast Co., Ltd., Tokyo, Japan) and water. 

Since ICR mice are superior to C57BL/6N mice in term of power balance, ICR mouse attacks a C57BL/6N mouse when they were placed in the same cage. CSDS model is established using the repeated aggressive behavior from ICR mice to C57BL/6N mice. C57BL/6N intruder mice were subjected to chronic social defeat stress using an ICR aggressor mouse as described previously [47,54] with minor modification. Once a day, the C57BL/6N mouse was subjected to defeat stress by exposure to the ICR aggressor mouse for 10 min. As a control, C57BL/6N mice were placed in the cage without an ICR aggressor mouse. This procedure was repeated for 10 consecutive days. All animal experiments in this study were approved by the Animal Use and Care Committee of Hyogo Medical University.

### 4.2. Social Interaction Test

**Figure 9:**
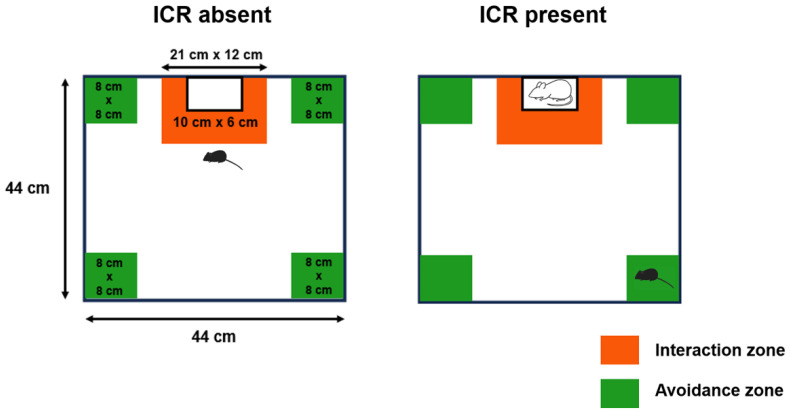
Social interaction test. C57BL/6N mice were placed in an open field chamber (44 cm × 44 cm) with or without an ICR aggressor mouse enclosed in a mesh cage (10 cm × 6 cm) for 150 s, respectively. The time that C57BL/6N mice spent in the avoidance zone and interaction zone was measured, respectively.

The social interaction test was performed as described previously [55], with minor modifications. In brief, C57BL/6N mice were placed in an open field chamber (44 cm × 44 cm) without an ICR aggressor mouse for 150 s. Thereafter, a mesh cage (10 cm × 6 cm) containing an ICR aggressor mouse was placed in the open field chamber, and the C57BL/6N mice were introduced for 150 s. The behaviors of the experimental mice were recorded for automated post-hoc analysis using the SMART video tracking system (Harvard Apparatus, Holliston, MA, USA). The time that the mice spent in the social interaction zone or the social avoidance zone was measured, respectively (Figure 9). The social interaction ratio (SIR) was calculated by dividing the time spent in the social interaction zone with the ICR mouse present by the time spent in the social interaction zone with the ICR mouse absent. In the present study, the mice were defined as susceptible to CSDS when the SIR was less than 50%, and only the mice susceptible to CSDS were used thereafter throughout the experiments.

### 4.3. Sampling of Stools and Intestinal Tissues

Fresh fecal samples from both the control and CSDS mice were collected after defecation on experimental day 11. The properties of the stools were scored according to the Bristol scale [24]. Fecal water content was assessed by the gravimetric drying method as previously described [56,57]. In brief, after weighing the wet stools, we dried them overnight at 65 °C and then determined their dry weight. The ratio of water in the stools was calculated as: (wet weight–dry weight)/wet weight. Then, under sevoflurane anesthesia, the mice were euthanized by cervical dislocation, and the small-intestinal tissues were removed. The small intestines obtained were cut open along the longitudinal axis, stored in nitrogen liquid for RNA extraction, or fixed in 10% buffered formalin solution for immunohistochemistry.

### 4.4. Analysis of the Gut Microbiome

In brief, bacterial DNA for sequencing was extracted from fresh stool samples using the phenol–chloroform method as described previously [58]. Analysis of the V3–V4 region of the microbial community 16S rDNA was amplified and sequenced using a 2 × 250-bp paired-end run on a MiSeq platform using MiSeq Reagent Kit v2 chemistry (Illumina, San Diego, CA, USA) as described previously [58]. Comparison of each taxon in the gut microbiota was conducted at both the genus and species levels. The Chao-1 and Shannon indices were calculated to assess the alpha diversity of the microbiota in the samples. Details of the methodology were described in the Section 4 of the reference paper [58].

### 4.5. Real-Time RT-PCR

Total RNA was extracted from small-intestinal tissues using Trizol reagent (Invitrogen, Carlsbad, CA, USA) in accordance with the manufacturer’s protocol and reverse-transcribed with an oligo-dT primer (Applied Biosystems, Branchburg, NJ, USA). Real-time RT-PCR was conducted using the 7900H Fast Real-time RT-PCR System (Applied Biosystems, Foster City, CA, USA) as reported previously [59]. Briefly, the sets of primers for mouse Claudin 4, Occludin, IL-1β, Regenerating gene IIIγ (Reg IIIγ), and GAPDH were prepared as shown in Supplementary Table S1. Real-time RT-PCR was performed with 200 ng of RNA equivalent cDNA, SYBR Green Master Mix (Applied Biosystems), and gene-specific primers at 500 nmol/l. The cycle conditions for real-time PCR were 50 °C for 15 s and 60 °C for 60 s. Relative mRNA levels of the target genes were normalized to the expression of GAPDH mRNA.

### 4.6. Histology and Immunohistochemistry

Immunohistochemical procedures were performed using an Envision kit (Dako, Kyoto, Japan) as described previously [47]. The primary antibodies used in this study were: anti-Occludin antibody (dilution 1:25, Invitrogen, Camarillo, CA, USA), anti-Reg IIIγ antibody (dilution 1:50; LSBio, Shirley, MA, USA), anti-lipopolysaccharide (LPS) antibody (dilution 1:50,000; Abcam, Cambridge, UK), and anti-CD80 antibody (dilution 1:1000; Abcam, Cambridge, UK). Briefly, the intestinal sections were incubated with primary antibodies for 60 min at room temperature, washed in PBS, and incubated with the corresponding secondary antibody for 30 min. The slides were visualized using 3,3′-diaminobenzidine tetrahydrochloride with 0.05% H_2_O_2_ for 3 min and then counterstained with Mayer’s hematoxylin. The number of LPS- or CD80-positive cells was evaluated as reported previously [60]. Briefly, four sections in each mouse were prepared for the small intestine. The positive cells were counted in at least five different visual fields in a 500 μm stretch of the entire length with well-oriented tissue sections, and the average was calculated as the number of positive cells per a villous in each mouse. The height of intestinal villi, the depth of intestinal crypt was evaluated using a microscope (DP72; Olympus, Tokyo, Japan) as previously reported [61].

### 4.7. Measurement of Transepithelial Electrical Resistance

Electrical resistance across the stratified epithelium was measured using a Millicell-ERS-2 instrument (Millipore, Bedford, MA, USA) with “chopstick” electrodes, as described previously [62]. Ex vivo permeability assays were performed as reported previously [63]. Briefly, the small-intestinal tissues were removed, opened longitudinally, placed apical side up on a 0.4 μm pore size membrane (Costar Incorporated, Corning, NY, USA), and transferred to a microsnapwell system. Electrical resistance was measured as described previously [62].

### 4.8. Statistical Analysis

Statistical analysis was performed using GraphPad Prism 8 (GraphPad, San Diego, CA, USA). Data are shown as mean ± SE. Significance of differences between two animal groups was analyzed by Mann-Whitney U-test. Differences were considered to be significant at *p* < 0.05.

## Data Availability

The datasets used and analyzed in this study are available from corresponding author.

## Supplementary Materials

The following supporting information can be downloaded at: https://www.mdpi.com/article/10.3390/ijms26199359/s1.

## Abbreviations

The following abbreviations are used in this manuscript:

CD80	Cluster of differentiation
CSDS	Chronic social defeat stress
ENS	Enteric nervous system
GAPDH	Glyceraldehyde-3-phosphate dehydrogenase
ICR	Institute of cancer research
IL	Interleukin
LPS	Lipopolysaccharide
Reg	Regenerating gene
RT-PCR	Reverse transcription polymerase chain reaction
SE	Standard error
TJPs	Tight junction proteins
