# Supplementary material for: Effect of Chronic Social Defeat Stress on the Small-Intestinal Environment, Including the Gut Flora, Immune System, and Mucosal Barrier Integrity
Source: Int J Mol Sci. 2025 Sep 25;26(19):9359. doi: 10.3390/ijms26199359 (PMC12525323; doi:10.3390/ijms26199359)
Supplement: Supplementary file 1 [file ijms-26-09359-s001.zip › ijms-3831369-supplementary.pdf]

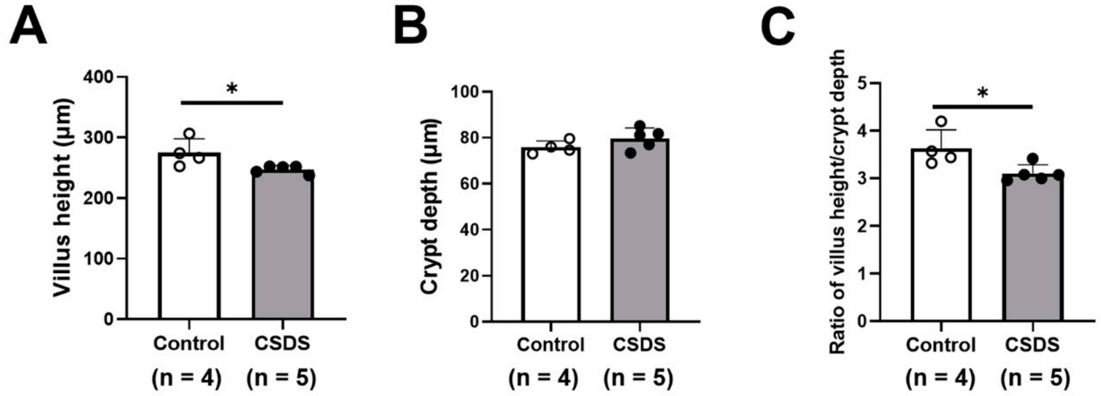

Supplementary Figure S1. Assessment of intestinal villi height (A), intestinal crypt depth (B) and ratio of villus height/crypt depth (C). * $p < 0.05$ vs. control group. CSDS, chronic social defeat stress.

Supplementary Table S1. Mouse primers for real-time reverse transcription-polymerase chain reaction analysis.

Genes	Direction	Sequences
Claudin 4	Forward	5’-GGAGGGCCTCTGGATGAACT-3’
	Reverse	5’-GATGCTGATGACCATAAGGGC-3’
Occludin	Forward	5’-TGAAAGTCCACCTCCTTACAGA-3’
	Reverse	5’-CCGGATAAAAAGAGTACGCTGG-3’
Il-1β	Forward	5’-TCCAGGATGAGGACATGAGCAC-3’
	Reverse	5’-GAACGTCACACACCAGCAGGTTA-3’
Reg IIIγ	Forward	5’-TTCCTGTCCTCCATGATCAAAA-3’
	Reverse	5’-CATCCACCTCTGTTGGGTTCA-3’
GAPDH	Forward	5’-GGAGAAACCTGCCAAGTATG-3’
	Reverse	5’-TGGGAGTTGCTGTTGAAGTC-3’
